# Functional Aspects of Seminal Plasma in Bird Reproduction

**DOI:** 10.3390/ijms21165664

**Published:** 2020-08-07

**Authors:** Julian Santiago-Moreno, Elisabeth Blesbois

**Affiliations:** 1 Department of Animal Reproduction, INIA, 28040 Madrid, Spain; 2 UMR-Reproduction Physiology and Behavior, INRAE, CNRS, IFCE, Tours University, 37380 Nouzilly, France; elisabeth.blesbois@inrae.fr

**Keywords:** avian species, cryopreservation, protein, lipids, sperm

## Abstract

This review provides an updated overview of the seminal plasma composition, and the role of metabolic and protein components on the sperm function of avian species. In addition, the implication of seminal plasma on assisted reproductive techniques of birds was discussed. The semen of birds usually has exceptionally high sperm concentration with relatively little seminal plasma, but this contributes to very fast changes in sperm metabolism and function. The biochemical characteristics and physiological roles of the various seminal plasma components in birds (carbohydrates, lipids, amino acids, hormones, and proteins) are poorly understood. Seminal plasma content of proteins has an action on most cellular functions: metabolism, immunity, oxido-reduction regulation, proteolysis, apoptosis, ion homeostasis, and antimicrobial defenses. The variable amount of many proteins is related to a different fertility capacity of poultry sperm. The role of seminal plasma on semen conservation (chilling and freezing) remains largely a matter of speculation, as both inhibitory and stimulating effects have been found. Whereas the presence of seminal plasma did not seem to affect the sperm survival after freezing–thawing, DNA fragmentation is lower in the absence of seminal plasma. The molecular basis of the influence of seminal plasma on sperm cryo-resistance was also discussed in the present review.

## 1. Introduction

Seminal plasma is a complex biological fluid that surrounds the sperm at ejaculation and shows crucial actions on semen biology in all animal species. The interaction of the seminal plasma with sperm induces metabolic changes, binding of seminal proteins onto the sperm cell surface and membrane remodeling, potentially affecting the sperm transport and storage in the female genital tract, and the zygote formation [1,2].

Birds represent an animal class with a highly specific reproductive process [3,4,5]. They are oviparous species with internal gonads, and gametogenesis conducted at a high body temperature (40–42 °C). They share a very complex system of internal fertilization involving the storage of sperm in specific sperm storage tubules (SST) of the female tract, and polyspermic fertilization of the big telolecithe oocyte. The male is the homogametic sex that usually produces, in a rapid spermatogenesis, a high number of sperm in very small volumes of semen; and post gonadic sperm maturation seems often not so clear. There is a lack of a copulatory organ in most bird species, but reproductive extra deferent annex glands (i.e, foam in quails) may show a huge impact on the fertilization success [6,7].

With all these specific features, bird seminal plasma compositions and roles may partly be common to all birds, and may partly be species-specific. They have mainly been approached through the study of domestic birds, especially chicken, and to a lesser extent the turkey [5,8,9,10,11,12]. A small number of studies focused on other domestic (i.e., Japanese quails, emu) or wild species (i.e., red jungle fowl, partridges, sparrows, budgerigar) [13,14,15,16,17]. In the last ten years, developments in biochemical and omics methods allowed for improving our knowledge of the seminal components and of the underlying mechanisms [12,18,19,20,21,22,23]. The presence of seminal plasma in the bird sperm in vitro storage media significantly influences the short- and long-term success of sperm conservation. If the full seminal plasma seems, in most cases, detrimental to long sperm storage, specific seminal fractions show clear protective actions [24,25,26,27], illustrating the complex relationships between the sperm cells and their environment.

In this review, after a rapid overview of the bird semen biology and of the seminal plasma composition, we will describe the known or expected roles of metabolic and protein actors of seminal plasma in avian species.

## 2. Bird Semen Biology

### 2.1. Semen Biology in the Male Tract

Bird spermatozoa are thin and long cells with a head containing a highly condensed genome in the nucleus, a small acrosome containing a number of enzymes needed for fertilization, and a vestigial perforatorium [28]. A mitochondrial sheet surrounding proximal and distal centrioles that are prolonged in a long flagellum in order to ensure spermatozoa motility follows this head. The number of mitochondria is extremely variable depending on the species: a mean of thirty in chickens and turkeys, up to 1000 in some quails. In this last case, many mitochondria are along the flagellum [28]. In most of the bird species that are polygamous, semen ejaculates are characterized by high mature sperm concentration (up to more than 10 billion/mL in the turkey), high sperm velocity and a low ratio of morphological abnormalities [28,29]. Unlike polygamous species, the existence of pleiomorphy with a high incidence of spermatocytes and spermatids are usual in monogamous species (e.g., eagles, falcons) [30]. The physiological role of these immature cells in the ejaculates is unknown, but they could be responsible for high levels of reactive oxygen species (ROS) in seminal plasma [30]. The semen of birds usually has exceptionally high sperm concentration with relatively little seminal plasma. The components of rooster seminal plasma derive from the proximal efferent ducts, epididymis and the deferent duct, including a terminal dilatation (receptaculum ductus deferentis) which enters the cloacum through an ejaculatory duct named papilla ductus deferentis [31,32]. There are no identified glands, such as prostate or seminal vesicles, in the avian deferent duct, unlike mammals. All these structures are intra-abdominal. Birds show a mean duration of spermatogenesis of 13 days in the chicken, followed by 1 day of transit of sperm in the extra-testicular ducts; the sperm survival time in vas deferens is ≤7 days. All these periods are shorter than in mammals: (about 38, 8 and 42 days, for spermatogenesis, epididymal transit and survival in epididymis and vas deferens, respectively) [31]. Although bird sperm cross the male ducts rapidly, these ducts resorb nearly 90% of the testicular plasma output [33]. The rapid spermatogenesis, about four times the rate of mammals, produces sperm that are almost mature [34]. The rapid transit of sperm through the extra-testicular ducts, and their short survival, contrast with the weeks that sperm are stored in the SST of the female reproductive tract [3,8]. During sperm transit from the testis to cloacum, the sperm present a series of changes as a result of their interaction with the components of extracellular medium produced by the epithelia of the genital tract. For example, one androgen-dependent protein (17 kDa) was identified in secretions of the epididymis of the Japanese quail [31]. Some proteins of the lower portion of the vas deferens bind to the surface of the sperm of Galliformes and will persist on the surface of spermatozoa in the female tract [35]. Early preliminary studies showed that sperm taken from the distal part of the deferent duct are much more able to fertilize than those taken from the testis or from the duct proximal to the testes in chicken [36].

### 2.2. Males Accessory Tissues and Extra Seminal Fluids

In birds, the male genital tract leads to the cloacum. In the species that do not share a specific appendix to transport the semen from the male to the female genital tract, an eversion of the cloacum ensures the contact between the male and the female genital tracts at mating. In the practice of artificial insemination, or for semen evaluation, semen is most often collected by dorso-abdominal massage, a method that is much less traumatic than electrical ejaculation with or without anesthesia [37]. In this practice, specific secretions of the male cloacum, such as the chicken “transparent fluid”, may contaminate the semen [8]. The composition of the chicken transparent fluid is close to lymphatic fluid. It is rich in glucose, a component often undetectable in chicken seminal plasma [38]. The high amounts of glucose of the transparent fluid stimulate sperm motility, but transparent fluid is deleterious to sperm in vitro storage.

Other products that transit through the cloacum, such as faeces and urine, may also contaminate the semen during semen collection. All these features imply a high technical skill when semen is collected by massage, in order to avoid any contamination of semen by the cloacum products. It is sometimes a difficulty in comparing the scientific studies of bird seminal plasma when they do not always have the same definition of seminal plasma.

Accessory sexual glands exist in specific species. This is the case of quails. During mating, the male quail adds milliliters of a white foam (while the volume of the ejaculate is often less than 20 µL) around the female vagina. The role of this foam is not fully elucidated. It has been reported to increase sperm motility [39] and to show contrasting effects on fertility [40]. However, more and more evidence indicates that this foam acts as a barrier to polyandric efficiency [6,7]. Indeed, it was proven that insemination of semen pooled from 3-6 males gave lower fertility results than inseminations of individual ejaculates.

In many duck species, a third situation exists. There are no accessory glands, and, at ejaculation, the semen is ejected through a specific gutter of the cloacum that is specifically unrolled at mating/massage [41]. In these species, the risk of contamination of seminal fluid by the cloacum fluid is much lower than in other species.

### 2.3. The Sperm in the Female Tract

Female birds are capable of prolonged sperm storage following mating or artificial insemination. Bird sperm are stored for several days or weeks in SST localized in the uterovaginal junction. Over the subsequent storage period the sperm are gradually released from the SST, ascend to the anterior end of the oviduct, and interact with next ovulated ovum [42]. The mechanisms of SST filling and emptying are not well known (sperm stratification, displacement of previous ejaculates or passive sperm loss [43]), but when a polyandrous female mates with multiple males (e.g., chicken, capercaillie, red-legged partridges), it is the sperm of the last male that usually fertilizes most of the eggs. Many seminal proteins involved in calcium metabolism [14] and mitochondrial functions [23], seem to influence the variation of sperm velocity. The velocity of sperm may involve the number of sperms that arrive at the SSTs, and thus securing last male precedence [44]. Moreover, sperm from low-mobility ejaculates tend to exit the SSTs sooner than fast-swimming sperm [45]. After sperm enter the SSTs, fluid secreted from the SST epithelial cells generates a current. Sperm with a good velocity might be able to move against this current and remain within the tubule [46].

## 3. Seminal Plasma Composition

### 3.1. Inorganic Ions

The seminal plasma contains the principal inorganic ions: Na^+^, K^+^, Cl^−^, Ca^2+^, and Mg^2+^. A reciprocal relationship exists between the account of Na^+^ and K^+^ in bird sperm and seminal plasma; seminal plasma contains higher Na^+^ and lower K^+^ than sperm. Chicken seminal plasma contains more Na^+^ and less K^+^ than in mammal species [32,47]. In chickens and turkeys, the most abundant inorganic ions are Na^+^ and Cl^−^; next to these ions, K^+^ is the most prevalent, and finally Ca^2+^ and Mg^2+^ are present in much lower concentrations [48,49].

### 3.2. Carbohydrates

Free carbohydrate analyses in seminal plasma from several chicken breeds showed that the inositol is the primary carbohydrate isolated (20.4 mg/dl) along with low amounts of glucose (3.1 mg/dl) and glycerol (2.8 mg/dl) [50]. The seminal plasma in poultries is characterized by very low (sometimes undetectable) concentrations of glucose [48], and is practically devoid of fructose [50]. Turkey sperm have a lower glycolytic activity than chicken sperm. The low concentrations of glucose could be related to the lack of glycolytic activity under anaerobic conditions in turkey [51]. The physiological roles of glucose in avian sperm are poorly understood. In chicken sperm, the ATP production via glycolysis plays a key role in the maintenance of flagellar motility and fertilization capacity [51,52]. A recent report suggested that glucose is involved in the regulation of the acrosome reaction in chicken sperm [53].

### 3.3. Lipids

The lipid composition of poultry seminal plasma (Table 1) varies significantly from the lipid content of sperm. The total lipid content of seminal plasma is lower than in the sperm [32], with a lower proportion of phosphatidylcholine [54], and high levels of cholesterol esters and triglycerides [9]. The species, breed, strains and the age may influence the total lipid content and the seminal plasma lipid profiles (free cholesterol, cholesterol esters, triglycerides, free fatty acids, phospholipids) [55,56]. Lipid concentrations in the seminal plasma of older roosters are greater than for younger roosters [55]. The major phospholipids in the seminal plasma of rooster are phosphatidyl choline and phosphatidyl ethanolamine. Phosphatidyl serine, phosphatidyl inositol, sphingomyelin and cardiolipin are found at a lower quantity [55]. The serine ethanolamine phosphodiester is found in avian seminal plasma. This non-cyclic phosphodiester appears to have a function as lysophospholipase inhibitors, decreasing the rate of membrane phospholipid turnover and lead to a net sparing of phospholipids [32]. The content of serine ethanolamine phosphodiester varies according to the age and the season [57]. The fatty acid composition of chicken seminal plasma includes a higher proportion of saturated fatty acids than sperm (49 vs. 39%) [58]. Polyunsaturated fatty acids (PUFAs) of the n-6 family, mainly arachidonic acid (20:4) and docosatetraenoic acid (22:4), are the major PUFAs routinely found in sperm and seminal plasma phospholipid of commercial chicken and turkey breeder strains [55,56,58]. However, much higher levels of n-9 unsaturated fatty acids were found in turkey semen compared to chicken. The n-3 PUFAs are usually found in far lower amount in both species. Their level may be increased by diet supplementation, allowing for increasing the semen fertilizing ability [58,59].

### 3.4. Hormones

Other organic compounds of the seminal plasma include steroid hormones, such as testosterone. Testosterone may be transported from rete testes fluid through the ductus deferent. The concentration of testosterone in seminal plasma was approximately two times less than in blood in both turkey [60] and chicken [61]. Seminal plasma testosterone concentration shows an inverse relationship with social status and blood plasma testosterone concentrations in red jungle fowl [62]. Testosterone concentrations in seminal plasma from ductus deferens and ejaculated seminal plasma are not significantly different [60,61].

### 3.5. Amino Acids

The seminal plasma amino acid profile may vary among genotypes, with strong differences between chicken breeds with respect to seminal free amino acid concentrations [63]. Glutamic acid is the most abundant free amino acid in seminal plasma, accounting for 76–89% (on molar basis) of the total amino acid content [63,64]. Glutamate is thought to serve as the main anion in place of Cl^−^ [65]. In addition, glutamate may act as a motility agonist when sperm is co-incubated with Ca^2+^ under aerobic conditions [46]; it has been suggested that rooster sperm express glutamate channels that mediate the flux of Ca^2+^ and K^+^ at the mitochondria membrane levels, hence contributing to sperm kinetics [46,66]. However, Santiago-Moreno et al. [63] did not find any relationship between glutamate and motility variables between breeds. Next to glutamine, the most abundant amino acids present in Mediterranean chicken breeds were alanine, serine, valine, and glycine (Table 2).

Some breeds show the same pattern of amino acids. The highest concentration of alanine, proline, cysteine and arginine are observed in the chicken breeds Black-red Andaluza, Birchen Leonesa, White-Faced Spanish and Quail Silver Castellana, respectively. The Buff Prat breed showed the lowest concentrations in these amino acids. Proline was relatively abundant in some breeds, but was below detection limits in other breeds [63]. Aspartic acid is the second most abundant amino acid in Delaware and New Hampshire roosters [67], and in Brown Leghorn [68]. Excluding glutamic acid, Ahluwalia and Graham [50] reported that arginine, asparagine, threonine, and glycine are the most prevalent in chicken seminal plasma of mixed breed. The other amino acids were present in much lower concentrations. Tryptophan is absent, or is present only in trace quantities.

### 3.6. Proteins

The seminal protein plasma concentration may vary among species and breeds [63]. Chicken seminal plasma contains 2.0–2.4 g/dl of protein [69,70], while in turkey, seminal plasma averages 1.8 g/dl [71]. In all cases, albumin comprises the majority of the protein content of poultry seminal plasma [72]. Many other seminal proteins have been identified. They include different enzymes, such as serine proteinase [73], phospholipases (A1 and A2), glycosidases (e.g., galactosidase, glucosidase, mannosidase, glucuronidase) [74,75], acid phosphatases [76], and a wide range of antioxidant enzymes [77,78]. The role of these and other proteins on bird sperm function is described below.

## 4. Role of Seminal Proteins and Peptides on Sperm Function

**Figure 1:**
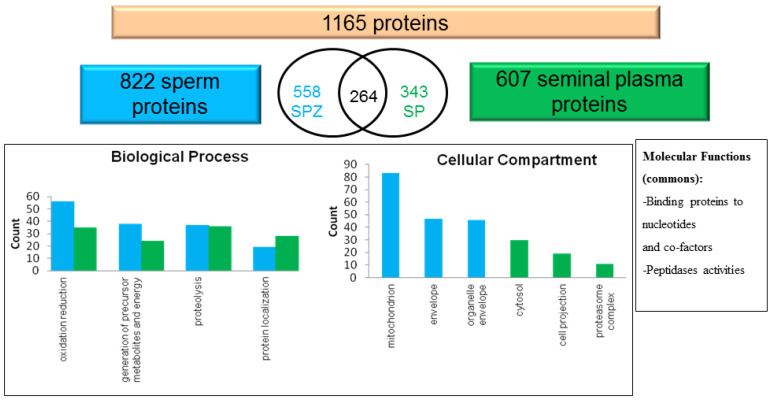
Respective composition of rooster plasma seminal and sperm proteins. Among the 1165 proteins observed by LC-MS, 22.6% were common to sperm (in blue) and seminal plasma (in green). The same general biological processes were found in both but cytosol products and components of proteasome were mainly extracted from seminal plasma [19].

In the last ten years, the emergence of without a priori proteomic studies offered new insights in the semen biology knowledge. Large proteome analyses (through liquid-chromatography-mass spectrometry (LC-MS) or intact-cell-Maldi-Tof mass spectrometry (ICM-MS) and tandem MS/MS), or targeted protein studies were performed in the chicken and the turkey. From all these studies, it appears clear that seminal plasma contains proteins expected to have an action on most cellular functions, and a significant part of them are common with the sperm cells, showing important exchanges between the sperm and their biological fluid (Figure 1).

They include different metabolic functions, immunity, oxido-reduction actors and regulation, proteolysis, apoptosis, ion homeostasis, antimicrobial defenses and also epigenetic actors such as histones [11,12,14,18,19,23].

### 4.1. Regulation of Membrane Stability and Fertilization Process

Since the birds’ sperm show small amounts of cytoplasm and a very high proportion of cellular and subcellular membranes (plasmatic, mitochondrial, nuclear, acrosome membranes), their lipid content is very high (mainly phospholipids) and demands extra cellular regulation through seminal components. Thus, enzymes regulating the lipids are active in seminal plasma. As an example, in turkeys, the phospholipase composition of seminal plasma is made to avoid premature sperm membrane destabilization, opposite to the oviductal fluid that prepares the sperm plasma membrane phospholipids bilayer destabilization, which contributes to initiating the fertilization process [10]. We expect that the same sort of regulation is present in other bird species.

**Figure 2:**
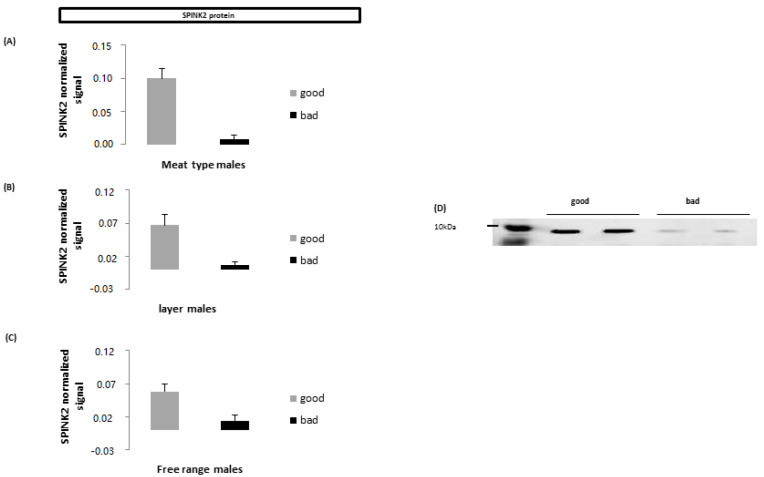
(**A**,**B**,**C**): respective seminal plasma intensity of the signal of SPINK2 protein in highly fertile males (grey) or in subfertile males (black: less than 40% fertility) [19] of 3 highly different lines of chicken (**A**): Meat line; (**B**): Laying line; (**C**): free range line. (**D**): western blott representation of differences of seminal SPINK2 amount in seminal plasma of highly fertile (good) and subfertile (bad) meat type males. Differences between “good” and “bad” males are highly significant in all cases. (Extracts from Thélie et al. [22]).

Since sperm are highly specialized cells, some seminal proteins are clearly turned to the regulation of specific sperm functions. This is the case of the regulation of enzymes involved in the preparation of fertilization and acrosome reaction expected to take place in the infundibulum of the female genital tract. When they are present in the seminal plasma, they can be regulated by seminal protease inhibitors. As an example, one of the enzymes important for fertilization liberated by the acrosome reaction is acrosin. The active form of acrosin was found in the seminal plasma of chickens and turkeys [19,23,73]. Acrosin is more abundant in the seminal plasma of subfertile sperm of roosters of highly different genetic background [19,22,23] and in submotile sperm of a Chinese local breed [23]. To avoid a premature activation of acrosin, the normal component expected to be found in the seminal plasma is the inactive proacrosin that can be cleaved in active acrosin under specific conditions. To limit the active acrosin content of seminal plasma, the seminal SPINK2 (serine peptidase inhibitor Kazal-type 2, also called Trypsin inhibitor CITI-1) is a serine proteinase inhibitor that shows a strong acrosin inhibition in the chicken [22]. Consequently, seminal acrosin is in higher content in chicken seminal plasma with low fertility, while SPINK2 is in higher content in the seminal plasma of semen with high fertilizing capacity in label, meat type and lay type breeds (Figure 2) [19,22].

### 4.2. Sperm Motility

Sperm motility shows species-specific differences. In birds, it was proven a long time ago that the presence of seminal plasma surrounding the sperm contributes to, and stimulates, the motility of sperm. In addition to other biochemical actors such as energetic substrates, protein components are involved in this regulation. For example, serum albumin, the most or one of the most abundant proteins of the chicken seminal plasma (depending on the studies/genetic background) stimulates chicken sperm motility at the normal concentrations found in seminal plasma [18,72]. This is also the case of ovalbumin [79], the main component of the egg yolk, also present in chicken seminal plasma, and probably of many other non-enzymatic proteins. The exact role of the bird seminal serum albumin has not been clearly defined, although we may expect that it is close to the seminal albumin role found in mammals. Seminal albumin is mostly secreted by the testis, the epididymis and the prostate in mammals, and a highly conserved function is suggested [80]. In mammals, seminal albumin is involved in the exchanges of lipids and other components between the sperm plasma membrane and its surrounding medium [81]. In birds, serum albumin and ovotransferrin are the most conserved proteins between the red jungle and the domestic chicken [11]. However, an abnormal and excessive high concentration of seminal albumin in turkey semen may reflect a subfertile phenotype named “yellow semen” [21].

Li et al. [23] found higher amounts of seminal SPINK2 in low motile sperm of the indigenous chicken breed Beijing-You. Considering that SPINK2 is an acrosin/trypsin inhibitor, protecting the sperm against damage by binding to the sperm membrane, these authors suggested that its greater abundance might be related to the failure of SPINK2 to bind to the membrane and acrosome of damaged sperm [23].

### 4.3. Antimicrobial Effect and Fertilizing Capacity

After the serum albumin, ovotransferrin is one of the most abundant proteins of chicken and of turkey seminal plasma [11,12,18,19]. Ovotransferrin is an important protein of egg yolk and shows large antimicrobial effects. We may expect that ovotransferrin shows the same role in bird seminal plasma. In contrast to the seminal albumin, ovotransferrin is not a relevant component of mammalian seminal plasma. A specific role of this component in the male and female bird reproduction might be suspected, possibly in relationship with a specific bird immunity system and antimicrobial defenses. Two other major proteins of the egg white showing strong antimicrobial and anti-proteolytic activity were described in chicken and turkey seminal plasma, the ovostatin and the ovoinhibitor [11,12,19]. Peptides involved in antimicrobial defenses were also described in the chicken seminal plasma, belonging mainly to the beta-defensins (Gallinacins) family [11,18,19]. One of them, the AVBD10, was more abundant in fertile chicken males than in subfertile males, leading the authors to suggest an important relationship between semen fertilizing capacity and antimicrobial defenses [19]. Other non-specific protein actors of the immune answer including proteins of the acute phase response pathway, or of the leukocyte activation and migration, or of the mediated immunity were described [12,14,18,19]. Taken together, these elements suggest that specific features of the male and female bird reproduction led to the implementation of specific antimicrobial barriers found in the semen and in the egg composition. Since it is clear that flying animals may cross highly different biotopes, we could hypothesise that strong antimicrobial defences in the reproductive system allow for greater safety in the production of healthy progeny.

### 4.4. Antioxidant Enzymes

Chicken, guinea fowl, common duck, European gander, and turkey seminal plasma are also enriched in antioxidant enzymes (i.e., Mn or Cu, Zn-SOD; Se or non Se-GSH; ceruloplasmin, paraoxonase, arylesterase) [77,78]. These enzymes contribute to the whole antioxidant effect of this complex medium, and by this route to the protection of the spermatozoa against the peroxidative action of the ROS [12,19,23,77]. However, the antioxidant defenses differ between species, relating to the respective activities of the anti-oxidant enzymes and, primarily, to the fatty acid composition of the semen of a given species, and to the capacity of aerobic and anaerobic metabolism. For example, since the turkey sperm seem to present exclusively aerobic metabolism [51], and do not take on anaerobic glycolysis, their demand for seminal antioxidants is higher than in the chicken.

### 4.5. Exosomal Proteins and Sperm Maturation

Some proteins, such as annexins, involved in calcium and signaling receptor binding, and functions such as apoptosis, would make up part of the still not clearly identified exosomes [14]. In mammals, different exosomes, such as epididymosomes, are important actors in sperm maturation through their constant exchanges with epididymal sperm [82]. They also have an action on the transfer to the progeny of epigenetic traits [82,83]. In birds, many protein components expected to make part of seminal exosomes were reported to be present in the seminal plasma of the domestic chicken [14,19,23] and of one of the chicken ancestors, the red jungle fowl [14]. They include members of the ras-related RB protein family, annexins and members of the 14-3-3 protein family. Seminal plasma and transparent fluid were not separated in the Borziak study [14], but we may expect that some of the components identified in this study make up part of the seminal secretions. We suggest that exosomes are present in bird as in mammalian semen, sharing many exchanges with the sperm and performing a modulatory effect on the post gonadic sperm maturation. A recent study [84] failed to highlight them, but the conditions of their extraction and observation seem to be particularly difficult in birds, and new advances in this area are expected in the next few years.

### 4.6. Lipoproteins and Fertility Expression

Some of the seminal proteins, such as apo-lipoprotein A1 (ApoA1), make up part of large molecular lipoprotein complexes such as high density lipoproteins and very high density lipoproteins involved in the extracellular transport of lipid components and in their exchanges with the sperm plasma membrane (HDL and VHDL) [9,10,24]. The ApoA1 content of chicken seminal plasma was present in differential amounts in chicken seminal plasma of males showing contrasted fertility capacity, increasing the suggestion of a key role of the lipoprotein complex of seminal plasma in the fertility expression [19].

### 4.7. Specie-Specific Proteins

In the two bird species that were the most studied (chicken and turkey), despite highly common seminal composition, there are also clear differences. One of them concerns the two proteins, HGFA (hepatocyte Growth Factor A) and CRISPs (cysteine rich secretory proteins), that seem abundant in turkey seminal plasma and lacking or minor in chicken [12], without any actual explanation. Since the role of these proteins is still unknown, we may only suggest that they refer to a species-specific function.

## 5. Implications of Seminal Plasma on Assisted Reproductive Technologies

Assisted reproductive biotechnologies involving bird semen mainly recover three situations [28]. One is artificial insemination with short semen in vitro storage, usually at room temperature (mainly in turkeys, guinea fowls, ducks interspecies hybrids, poultry selection). A second case is longer in vitro semen storage with chilling (for semen transportation, length duration up to 24 h). The third case is sperm cryopreservation involved in conservation programs implying many years of semen storage. The role of seminal plasma is important in these three situations since it is still usually present in semen and all its components may be very active in vitro. The role of bird seminal plasma shows common features for these three methodologies, but also shows actions that are more predominant in specific technologies. In all of these technologies, the intense sperm metabolism and the high proteolytic activities of seminal enzymes imply very rapid dilution of bird semen in more “neutral” diluents (usually saline diluents enriched with glucose or fructose) in order to avoid an extremely rapid degradation of sperm in vitro. Indeed, fresh undiluted sperm lose their fertilizing capacity in less than 1h in chicken, and less than 30 min in guinea fowl or turkey [3,28]. A decrease in the semen temperature from 41 °C to a mean of 20 °C is helpful for in vitro storage of less than one hour; and a decrease to 4–5 °C is needed for longer storage without cryopreservation. These decreases in temperature reduce the seminal enzymes’ activities, the metabolism intensity and the production of end by-products potentially harmful to sperm (i.e., peroxides, excess of lactic acid...). During all types of in vitro storage, the sperm may actively use the metabolic by-products present in seminal plasma (i.e., in birds, fatty acids or amino acids…) in order to allow the preservation of all sperm cellular compartments, and to ensure the conservation of sperm motility.

The semen cryopreservation process shows specific added features since the freezing, and then the thawing process induces very high osmotic and thermic stresses, and the potential presence of extra- and intracellular ice formation. These stresses increase the physical constraints applied to the cells, but also the oxidations stress induced by ROS produced during the process [85]. Consequently, the sperm membrane composition may be severely affected. For example, the fatty acid composition of frozen-thawed chicken sperm shows a reduction in PUFAs and an increased proportion of saturated fatty acids [86]. In this situation, the role of the ions and biochemical components of the seminal plasma may have different impacts (pro- or antioxidant, protection of the cell membranes, etc.).

The role of seminal plasma in bird semen in vitro storage remains largely a matter of speculation as both inhibitory and stimulating effects have been found.

The seminal plasma may contain factors that prevent cryoinjury, but also components with detrimental effects on sperm preservation. Previous reports showed contrasting effects of seminal plasma fractions on chilled rooster sperm [25], and a global deleterious effect in chickens and turkeys [24,27]. Low molecular weight seminal plasma fractions would reduce the fertilizing ability of sperm during storage at 4 °C, whereas high molecular weight fractions appeared to enhance fertilizing ability [25]. The presence of high zinc concentration in seminal plasma had a detrimental effect on the fertilizing ability of stored spermatozoa for 24 h at 4 °C [87]. Seminal plasma is involved in the degradation of sperm phospholipids, possibly by phospholipase activity, accelerating the sperm damage of turkey sperm during in vitro storage [27]. On another hand, the high antioxidant defenses of seminal plasma [77] may be involved in protective actions during in vitro storage. In addition to antioxidant enzymes and vitamins, seminal amino acids seem to play an active role in this area.

Specific seminal plasma amino acids are associated with post-thaw percentage of viable sperm and DNA integrity (Table 2). Negative correlations have been found between sperm DNA fragmentation after freezing and the concentrations of the following amino acids in seminal plasma: valine, alanine, isoleucine, methionine, leucine, tyrosine, phenylalanine, serine, most of which are of hydrophobic nature [63]. Although many of the cryoprotectant mechanisms of free amino acids are not well known, their role on sperm cryoresistance appears to be related to their antioxidant activity as recently shown for valine and serine [63,88], by serving as oxidizable substrates for scavenge free radicals. Furthermore, amino acids may provide protection against the denaturing effects of low water potential during freezing [89]; amino acid might stabilize proteins, thus avoiding denaturation and dissociation that would lead to a greater contact surface between proteins and solutes during freeze-thaw process [90]. Amino acids may interact with phospholipids bilayers during freezing [91], allowing for stabilizing the cell membrane. Heber et al. [92] noted an amino acids protective action on sperm during freezing by colligative action, through their unspecific ability to reduce the concentration of toxic solutes below the limit of toxicity. In addition, amino acids may provide buffers with a protective influence on sperm cells [47].

Several studies have focused on the use of amino acid as additives in extenders. Accordingly, supplementing chicken extenders with the non-coded amino acid taurine showed a positive effect in reducing sperm apoptosis and DNA damage [93]. Supplementation of valine to chicken freezing extenders showed a positive effect on DNA fragmentation and fertilizing ability of frozen-thawed sperm, with a better response in a breed considered as the lowest freezer [94]. The addition of appropriate serine levels also showed a positive effect on sperm cryopreservation, partly through the antioxidant action of this amino acid [88].

Glutamine, proline, histidine, glycine, and alanine have been used as additives in semen extenders in many species [95,96,97,98]. Glutamate, the main amino acid of bird seminal plasma, is usually used as a component of the freezing medium to improve the sperm motility [99]. It also contributes greatly to the maintaining of the seminal plasma osmolality. Some amino acids, such as proline, might act as a solute protecting the cell against the denaturing effects of hyperosmolality induced by dehydration during slow freezing [89].

In contrast, seminal cysteine concentration was reported to be negatively correlated with DNA integrity in fresh samples of native chicken breeds [63]. There is not information about the use of this amino acid in avian semen, but the supplementation of extenders with L-cysteine for the cryopreservation of carp sperm reported a decrease in DNA damage in post-thawed sperm [100].

**Figure 3:**
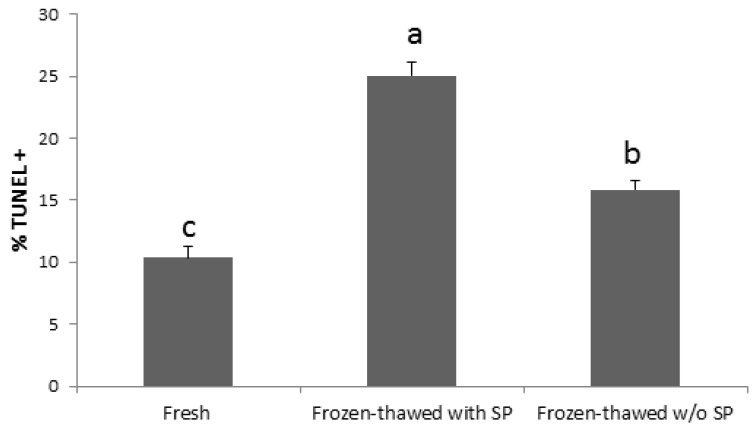
Percentage of chicken sperm TUNEL + (DNA fragmentation assessed by terminal deoxynucleotidyl transferase dUTP nick end labelling) in fresh sperm and after freezing-thawing samples (*n* = 90) with or without (w/o) seminal plasma. Different letters (a, b, c) indicate significant differences (*p* < 0.05).

Whereas the presence of seminal plasma did not seem to affect the rooster sperm survival after freezing and thawing, the DNA was less damaged without plasma (Figure 3) [63].

DNA appears to be more sensitive to the generation of ROS [101], and it has been suggested that ROS production is stimulated by the presence of seminal plasma. The overproduction of ROS, which exceeds the seminal plasma antioxidant capacity, disturbs the balance between seminal ROS and antioxidant capacity, and results in oxidative stress. Oxidative damage may originate from several potential resources from seminal plasma, such as leukocytes [102] and the presence of immature sperm. The presence of spermatocytes and spermatids in semen is one characteristic of falcon ejaculates, and strongly affects sperm in vitro storage (chilling and freezing) by generating excessive amounts of ROS themselves [30]. Other non-identified components of the seminal plasma of birds might also stimulate excessive generation of ROS by sperm and/or strongly decrease the levels of antioxidant defenses [103] during freezing–thawing process. Given the above information, the removal of seminal plasma previously freezing in chicken semen may be a suitable strategy to reduce DNA damage of sperm [63]. Alternatively, the development of selective washing techniques (e.g., density-gradient centrifugation) in falcon sperm might be a useful strategy to eliminate immature cells and to decrease ROS production. Conversely, in other wild species studied in our lab, such as American flamingo (*Phoenicopterus ruber*), seminal plasma removal does not provide a benefit for sperm cryopreservation [104].

## 6. Conclusions

As in other animal species with sexual reproduction, bird seminal plasma is a complex fluid involved in key exchanges with sperm, which greatly influence the fertilizing capacity of the gametes. The semen volume is usually low and the sperm concentration very high in birds, thus the seminal plasma is in a relative low amount, but it contributes to very fast changes in sperm metabolism and functions. Seminal plasma is eliminated rapidly in vivo in the bird female vagina after mating, but in the in vitro semen biotechnologies, its presence shows a great impact on the success of bird sperm fertilizing capacity.

## Figures and Tables

**Table 1:** Lipid composition of seminal plasma from chicken and turkey [9,55,56].

**Lipid**	**Chicken**	**Turkey**
Total lipid (µg mL^−1^ seminal plasma)	623.4	124
Phospholipid (% of total lipids)	31.3	50.7
Free cholesterol (% of total lipids)	18.0	31.1
Triglycerides (% of total lipids)	21.1	6.8
Free fatty acid (% of total lipids)	13.7	2.5
Cholesterol ester (% of total lipids)	15.9	12.3
**Phospholipids**		
Phosphatidyl choline (% of total phospholipids)	15.5	4.6
Phosphatidyl ethanolamine (% of total phospholipids)	43.6	31.5
Sphingomyelin (% of total phospholipids)	13.4	20.3
**Lipid**	**Chicken**	**Turkey**
Total lipid (µg mL^−1^ seminal plasma)	623.4	124
Phospholipid (% of total lipids)	31.3	50.7
Free cholesterol (% of total lipids)	18.0	31.1
Triglycerides (% of total lipids)	21.1	6.8
Free fatty acid (% of total lipids)	13.7	2.5
Cholesterol ester (% of total lipids)	15.9	12.3
**Phospholipids**		
Phosphatidyl choline (% of total phospholipids)	15.5	4.6
Phosphatidyl ethanolamine (% of total phospholipids)	43.6	31.5
Sphingomyelin (% of total phospholipids)	13.4	20.3

**Table 2:** Seminal plasma free amino acid concentrations (mM) from chicken. (Mean, Standard deviation: SD) and their correlation with sperm viability (C:VB) and DNA integrity (C:DNA) after freezing-thawing.

	Glu	Ala	Ser	Val	Gly	Thr	Pro	Asp	Leu	Arg	Ile	Phe	His	Cys	Met	Tyr	Lys
Mean	44.9	1.3	1.2	0.9	0.9	0.7	0.6	0.5	0.5	0.5	0.3	0.3	0.3	0.2	0.2	0.2	0.2
SD	8.3	0.3	0.3	0.2	0.2	0.2	0.1	0.1	0.1	0.2	0.1	0.1	0.1	0.1	0.1	0.1	0.1
C:VB				*					*		*						*
C:DNA		*	*	*					*		*	*			*	*	

Glutamic acid (Glu), alanine (Ala), serine (Ser), valine (Val), glycine (Gly), threonine (Thr), proline (Pro), aspartic acid (Asp), leucine (Leu), arginine (Arg), isoleucine (Ile), phenylalanine (Phe), histidine (His), cysteine (Cys), methionine (Met), tyrosine (Tyr), lysine (Lys). (*n* = 48 samples from Mediterranean chicken breeds). Asterisks indicate amino acid positively correlated (*p* < 0.05) with sperm viability (evaluated by propidium iodide/SYBR-14) and DNA integrity (evaluated by TUNEL).
